# Partial nerve injury induces electrophysiological changes in conducting (uninjured) nociceptive and nonnociceptive DRG neurons: Possible relationships to aspects of peripheral neuropathic pain and paresthesias

**DOI:** 10.1016/j.pain.2012.04.019

**Published:** 2012-09

**Authors:** Laiche Djouhri, Xin Fang, Stella Koutsikou, Sally N. Lawson

**Affiliations:** School of Physiology and Pharmacology, University of Bristol, Bristol BS8 1TD, UK

**Keywords:** Uninjured neuron, Spontaneous firing, Dorsal root ganglion, Nociceptor, Pain behaviour, Nerve injury, Nociception, Neuropathic pain, Threshold, Allodynia, Hyperalgesia, Neuroinflammation, Spontaneous pain, Allodynia, Hyperalgesia, Paresthesia, Action potential, Membrane potential, In vivo, Intracellular recording, Aβ-nociceptors, A-fibre nociceptors, C-fibre nociceptors, Low-threshold mechanoreceptors

## Abstract

Partial nerve injury leads to peripheral neuropathic pain. This injury results in conducting/uninterrupted (also called uninjured) sensory fibres, conducting through the damaged nerve alongside axotomised/degenerating fibres. In rats seven days after L5 spinal nerve axotomy (SNA) or modified-SNA (added loose-ligation of L4 spinal nerve with neuroinflammation-inducing chromic-gut), we investigated a) neuropathic pain behaviours and b) electrophysiological changes in conducting/uninterrupted L4 dorsal root ganglion (DRG) neurons with receptive fields (called: L4-receptive-field-neurons). Compared to pretreatment, modified-SNA rats showed highly significant increases in spontaneous-foot-lifting duration, mechanical-hypersensitivity/allodynia, and heat-hypersensitivity/hyperalgesia, that were significantly greater than after SNA, especially spontaneous-foot-lifting. We recorded intracellularly *in vivo* from normal L4/L5 DRG neurons and ipsilateral L4-receptive-field-neurons. After SNA or modified-SNA, L4-receptive-field-neurons showed the following: a) increased percentages of C-, Ad-, and Ab-nociceptors and cutaneous Aa/b-low-threshold mechanoreceptors with ongoing/spontaneous firing; b) spontaneous firing in C-nociceptors that originated peripherally; this was at a faster rate in modified-SNA than SNA; c) decreased electrical thresholds in A-nociceptors after SNA; d) hyperpolarised membrane potentials in A-nociceptors and Aa/b-low-threshold-mechanoreceptors after SNA, but not C-nociceptors; e) decreased somatic action potential rise times in C- and A-nociceptors, not Aa/b-low-threshold-mechanoreceptors. We suggest that these changes in subtypes of conducting/uninterrupted neurons after partial nerve injury contribute to the different aspects of neuropathic pain as follows: spontaneous firing in nociceptors to ongoing/spontaneous pain; spontaneous firing in Aa/b-low-threshold-mechanoreceptors to dysesthesias/paresthesias; and lowered A-nociceptor electrical thresholds to A-nociceptor sensitization, and greater evoked pain.

## Summary

After partial nerve injury, changes in uninjured nociceptors and nonnociceptors (spontaneous firing, decreased electrical thresholds, hyperpolarisation) may account for the different aspects of neuropathic and paresthesias.

## 1 Introduction

Neuropathic pain of peripheral origin (NP) often results from partial injury of a peripheral nerve resulting in dorsal root ganglion (DRG) neurons with axotomised (nonconducting), and uninterrupted fibres, able to conduct from their receptive fields (RFs). Although the latter are often called uninjured, they may be altered. NP is characterized in humans by spontaneous pain, and/or evoked pain resulting from hypersensitivity to normally painful stimuli (hyperalgesia) or normally nonpainful stimuli (allodynia) [12]. This pain may be accompanied by peculiar sensations, dysesthesias, and/or paresthesias [6]. For increased somatosensory pain to arise from the periphery, DRG neurons must supply increased information to the central nervous system (CNS). Changes in the CNS may contribute, driven, at least in part by increased afferent input [15,16,18,46,60,64]. In a partially injured somatic nerve, degeneration of axotomised fibres causes neuroinflammation (raised trophic factors, cytokines, inflammatory mediators) [47,59,75]. The relative contributions to NP of DRG neurons with axotomised fibres [15], or with uninterrupted/conducting fibres, are far from clear.

The uninterrupted fibres of these DRG neurons with RFs (RF-neurons) are influenced by the neuroinflammation. Some RF-neurons show spontaneous firing (SF) including both C- and A-fibre neurons [13,21,77]. There are growing and compelling arguments for a major contribution of these RF-neurons with uninterrupted/conducting fibres to NP, including 1) SF in these, but not axotomised, nonregenerating, C-fibre neurons [13,21,50,51,77]; 2) the necessity of such afferent fibres for sensory stimulation-evoked firing to reach the CNS; and 3) sensitization of such nociceptors to sensory mechanical stimulation [66].

Likely mechanisms of increased afferent input in NP include increased SF, and decreased thresholds, resulting in greater firing to noxious or innocuous stimuli in neurons with uninterrupted/conducting fibres, but the full extent of changes in these DRG neurons after partial nerve injury is unknown.

Furthermore, in order to understand contributions of RF-neurons with uninterrupted fibres to different aspects of NP, it is important to determine which sensory neuronal subtypes change their electrophysiological properties after nerve injury. Differing central projections [31] of different sensory subtypes probably influence the nature of resulting pain/sensations. Several previous electrophysiological studies examined changes in properties of L4 neurons after L5 spinal nerve injury [13,21,53,63,77,80], but none (including our previous study [21]) was limited to RF-neurons. This is important because fibres of some L4 neurons are damaged during L5 spinal nerve surgery [13,21,77]. Therefore, L4 neurons recorded in the NP models in the present study are limited to RF-neurons, that is, with physiologically identified receptive properties.

We made intracellular recordings in vivo in normal L4/L5 DRG neurons in normal (untreated) rats and in L4 DRG RF-neurons in 2 rat models of NP. These were L5 spinal nerve axotomy (SNA), and modified SNA (mSNA) with additional loose ligature of the L4 spinal nerve with chromic gut, which induces neuroinflammation [54,68]. Use of both models enables study of effects of additional neuroinflammation in mSNA on L4 RF-neurons. This study provides novel information about electrophysiological/membrane changes in different subgroups of L4 RF-neurons in vivo that are likely to result in increased CNS input and thus contribute to pathological pain or altered sensation.

## 2 Materials and methods

### 2.1 Animals and in vivo preparation

All experimental procedures (ie, surgery and recording) were carried out under deep anaesthesia (sodium pentobarbitone 60 mg/kg intraperitoneally) on young adult female Wistar rats (150–180 g). All procedures were performed under a licence held under the provision of the UK Animals (Scientific Procedures) Act 1986, and reviewed by the University of Bristol Ethical Review Group. They comply with the policies and recommendations of the International Association for the Study of Pain. At the end of the experiments, animals were killed with an overdose of anaesthetic. The 2 models of neuropathic pain of peripheral origin described below were described previously [21] and are modifications of the original Chung spinal nerve ligation (SNL) model [44].

**Fig. 1:**
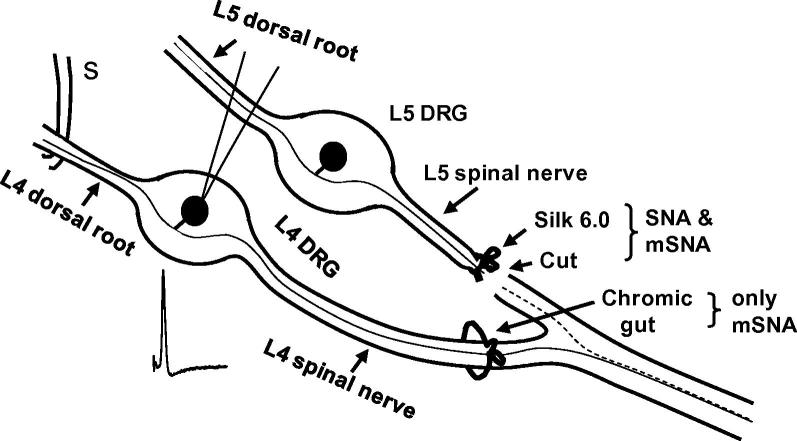
Diagram of models: The L5 spinal nerve was ligated (with 6.0 silk suture) and transected in both spinal nerve axotomy (SNA) and modified SNA (mSNA) models; the L4 spinal nerve was loose-ligated with chromic gut in mSNA only. The dashed and continuous lines in the sciatic nerve indicate degenerating axotomised L5 fibres and adjacent uninterrupted L4 fibres, respectively. Electrophysiological properties recorded from uninterrupted L4 dorsal root ganglion (DRG) receptive field-neurons in both models 7 days after surgery were compared with those from L4 and L5 DRG neurons in normal rats. Somatic action potentials were evoked by dorsal root electrical stimulation (S).

Three groups of animals were used: 1) *Normal (untreated) group:* This group of animals had no prior surgery; data from normal L4 and L5 DRG are labelled in figures as L4/5 Norm; 2) *SNA (L5 Spinal Nerve Axotomy) group:* An incision above the lumbar spine exposed the left transverse process of the L6 vertebra, which was then removed. The L5 spinal nerve was then isolated, tightly ligated with a 6-0 silk suture and transected (to prevent fibre regeneration) just distal to the suture, with care to minimize damage to the L4 spinal nerve (Fig. 1). The skin incision was closed with intracutaneous sutures, and healing occurred normally in all cases by day 7 after surgery; 3) *mSNA (modified SNA) group:* in addition to the above L5 SNA, the L4 spinal nerve was ligated loosely with a 5.0 chromic-gut suture (5-0; Ethicon, Livingston, Scotland, UK; and LOOK, Angiotech, Vancouver, BC, Canada), making a loop at least 2 mm greater than the diameter of the nerve (see [21,49] and Fig. 1), or much looser than that used in the chronic constriction injury model [9]. The chromic gut causes neuroinflammation, and the physical presence of the ligature may also cause some mechanical damage [21,49]. Data from the adjacent ipsilateral L4 DRG RF-neurons with conducting fibres in SNA and mSNA models are labelled in figures as SNA and mSNA. The surgery for both models was carried out under sterile conditions.

We did not use a separate sham-operated group because it was previously shown that electrophysiological properties of DRG neurons after sham L5 SNL operations were similar to those in unoperated normal rats [53], and it was therefore not justifiable to use a further group of sham-operated rats. Furthermore, any nerve injury due to sham operation would contribute to the changes under observation.

### 2.2 Nomenclature of models

The acronym SNL was used in the original description of the spinal nerve injury model [44] to mean spinal nerve tight ligation. We use the terms SNA and mSNA because in these models, the L5 spinal nerve is axotomised [21]. It is important to distinguish between SNA vs SNL and mSNA vs mSNL, because there is greater and more reliable fibre damage after L5 SNA than L5 SNL, and we have evidence that behavioural signs of both mechanical hypersensitivity and spontaneous pain are significantly greater in the mSNA than mSNL models (Koutsikou and Lawson, in preparation).

### 2.3 Pain behavioural tests and observations

**Fig. 2:**
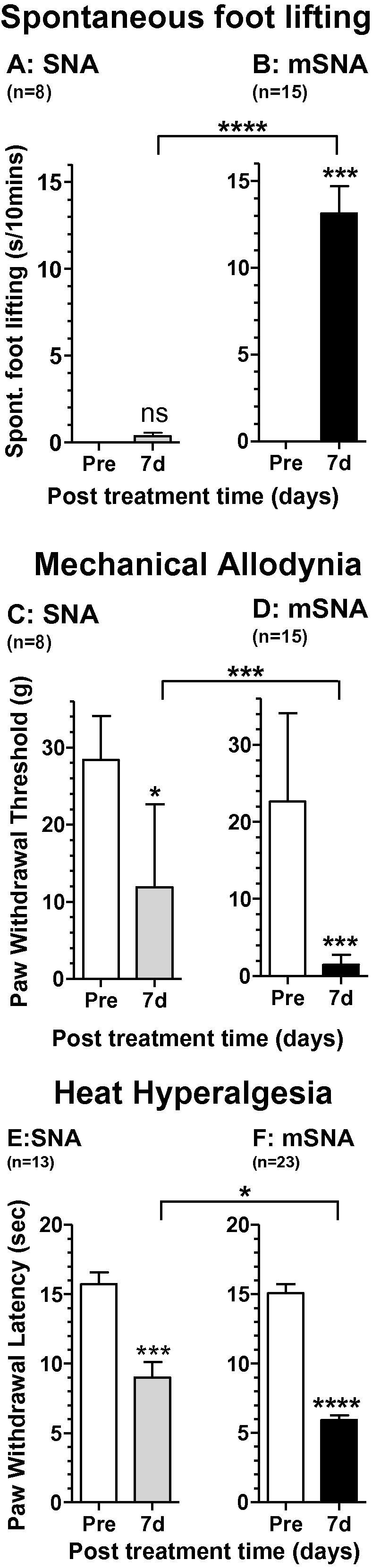
Neuropathic pain behaviour for spinal nerve axotomy (SNA) and modified SNA (mSNA): for each behavioural type, comparisons were between preoperative levels (open bars) and 7-day behaviour for SNA (grey) and mSNA (black bars). Tests were paired *t*-tests for A and B, and Wilcoxon matched-pairs signed rank test for C to F. There was greater spontaneous foot lifting (A), mechanical allodynia (B), and heat hyperalgesia (C) in mSNA than SNA, but no difference between the behaviours presurgery between SNA and mSNA groups (Mann-Whitney tests).

Studies were made in 23 rats (8 SNA and 15 mSNA) for all 3 behaviours described below, with additional rats for heat hypersensitivity (see Fig. 2). Tests were made in a chamber with clear plastic walls between 8 and 11 am. After acclimatisation to tests and chambers, tests were performed 1 day prior to surgery (referred to as Pre in the bar plots) and 7 days postoperatively. For each test, rats were allowed time to acclimatise in the chamber until exploratory and grooming behaviour ceased (>10 minutes). Stimuli were applied to the mid-plantar surface of the hind paw (L4 dermatome), avoiding the footpads. As described previously [21], behavioural testing could not be performed blindly because of deformation of the ipsilateral foot in the SNA and mSNA rats.

#### 2.3.1 Spontaneous foot-lifting

Spontaneous foot-lifting (SFL) duration has been used as a measure of spontaneous/ongoing pain [1,7,9,21,43,52,65]. It was measured as the cumulative duration of time that the rats lifted (sometimes followed by shaking and/or licking) their ipsilateral hind foot off the glass floor over 10 minutes (2 5-minute periods separated by ⩾5 minutes). No SFL was observed contralaterally or in normal rats. Foot-lifting associated with locomotion, body repositioning, or grooming [43,79] was excluded. SFL was measured in stationary rats and as reported previously [21]; there was no correlation between SFL and static (von Frey) mechanical allodynia in mSNA rats [21]. Thus, SFL was not due to mechanical allodynia.

#### 2.3.2 Von Frey hair-evoked withdrawal threshold

This test is used to determine mechanical-hypersensitivity/mechanical allodynia [17,43]. Each hind paw was touched perpendicularly for ∼5 seconds through a metal mesh floor, with one of a series of 6 von Frey hairs [17]. Paw withdrawal during von Frey hair application or on its removal was recorded. The 50% withdrawal threshold was determined with the up-and-down method [17].

#### 2.3.3 Withdrawal latency to noxious heat

A plantar (Hargreaves) Analgesy-Meter (Ugo Basile, Italy) was used to quantify heat hypersensitivity (heat hyperalgesia). The average was taken of 3 latencies for each hind paw measured with ⩾5-minute intervals.

### 2.4 Intracellular electrophysiological recordings

Under deep anaesthesia (Na^+^ pentobarbitone anaesthesia, see above), recordings were made 7 days postoperatively or in normal rats of similar age/weight. Full details of the animal preparation were as reported previously for guinea pig [22] and rat [28]. Briefly, a tracheotomy allowed artificial ventilation and monitoring of end-tidal CO_2_, which was maintained between 3% and 4% by adjusting the rate and/or volume of the respiratory pump. The left jugular vein and carotid artery were cannulated to enable, respectively, regular injections of anaesthetic and monitoring of blood pressure (normally ∼80–100 mm Hg). The left hind limb was extended and fixed (plantar surface upwards).

After a laminectomy from vertebrae L2-L6, the L4 and L5 DRG (normal) or L4 DRG (SNA, mSNA) and their corresponding dorsal roots were exposed and covered with warmed (30°C) paraffin oil in a large pool constructed using dental impression material. To improve recording stability during recording, all animals were given a muscle relaxant (pancuronium, 0.5 mg/kg intravenously) prior to recording, which was repeated approximately hourly, and was always accompanied by an additional dose (10 mg/kg, intravenously) of anaesthetic. This amount and frequency of anaesthetic administration during the period of muscle relaxant was the same as that required to maintain complete areflexia (lack of limb withdrawal reflexes to noxious stimulus) in the absence of muscle relaxant during the preceding 2-hour surgery period. The temperature near the DRG in the paraffin pool was maintained close to ∼30° (range 28–32°C) and the core temperature maintained at ∼34–35°C. Recordings were made online with a CED (Cambridge Electronics Design, Cambridge, UK) 1401plus interface and spike II programs from CED.

Intracellular voltage recordings from neuronal somata were obtained using sharp glass microelectrodes. These recording electrodes were filled with KCl (1 M or 3 M, median resistance 74 MΩ) for all recordings of membrane potential (Em), action potential (AP), and for some of the SF and threshold records. To increase the amount of SF and threshold data, records made for a different study with higher resistance electrodes filled with 0.1 M LiCl plus Lucifer Yellow dye [28] were also included. All records were made before Lucifer Yellow was ejected, and since the electrical threshold was measured in response to electrical stimulation of the L4 dorsal root (see below), that is, distant from the DRG, it would not be influenced by electrode content. Neither threshold values nor incidence of SF differed between recordings made with KCl- and those with LiCl-filled electrodes.

### 2.5 Electrophysiological variables

#### 2.5.1 Membrane potential (Em) and action potentials (APs)

Em was recorded after it had stabilized following initial penetration, and at the time of somatic AP recording. Somatic APs were antidromically evoked by dorsal root electrical stimulation with bipolar platinum electrodes, using single rectangular pulses (0.03 ms duration for A-fibre units or 0.3 ms for C-fibre units). The stimulus intensity was adjusted to twice threshold for A-fibre units and between 1 and 1.5 times threshold for C-fibre units. APs were analyzed offline using the CED Spike II program and scripts as previously described [21].

#### 2.5.2 Electrical threshold

This was the minimum stimulus (in volts) applied to the dorsal root that evoked a somatic AP. Occasionally, tissue fluid accumulates around the nerve, which increased the voltage required; this fluid accumulation was therefore minimized by constant checking and regularly wicking the fluid away. Any accumulation would have increased variability to a similar extent in experimental and normal groups, thus, the direction of change of medians is clear, even if some values are overestimated.

Although C-fibre neurons are encountered frequently during intracellular recording, they are much more difficult to penetrate successfully, and maintain stable recordings in, than A-fibre neurons. Indeed, many C-fibre neurons were lost before recording was completed, and are thus not included in this paper. Because of this, and the need to restrict to a minimum the number of high-voltage stimuli applied to the dorsal root, the electrical threshold was not accurately measured for most C-fibre units. C-nociceptor electrical thresholds were therefore not included, although they were clearly much higher than for A-fibre neurons.

#### 2.5.3 Conduction velocity

Conduction velocity (CV) was determined by dividing the conduction distance (4.5–14 mm, typically about 10 mm) by the latency to onset of the evoked somatic AP. This latency was measurable even for Aα/β-fibres (as can be seen later in Fig. 4), but with less accuracy in very rapidly conducting Aα/β-neurons when measured over the shortest conduction distances.

Neurons were classified according to their dorsal root CVs as C (⩽0.8 m/s), Aδ (1.5–<6.5 m/s), or Aα/β (⩾6.5 m/s). These borderlines were defined from compound AP recordings from L4/L5 dorsal roots of female rats of the same age/weight and at the same pool temperature [28]. They were relatively low, for reasons including use of the dorsal root for CV measurements [74], the pool temperature (∼30°C), and inclusion of utilization time. For further details see [23]. Only 1 C/Aδ-neuron (CV between 0.8 m/s and 1.5 m/s) was recorded in the normal group, and none in the mSNA or SNA groups. This neuron was therefore excluded.

#### 2.5.4 Spontaneous firing (SF) recordings

**Fig. 3:**
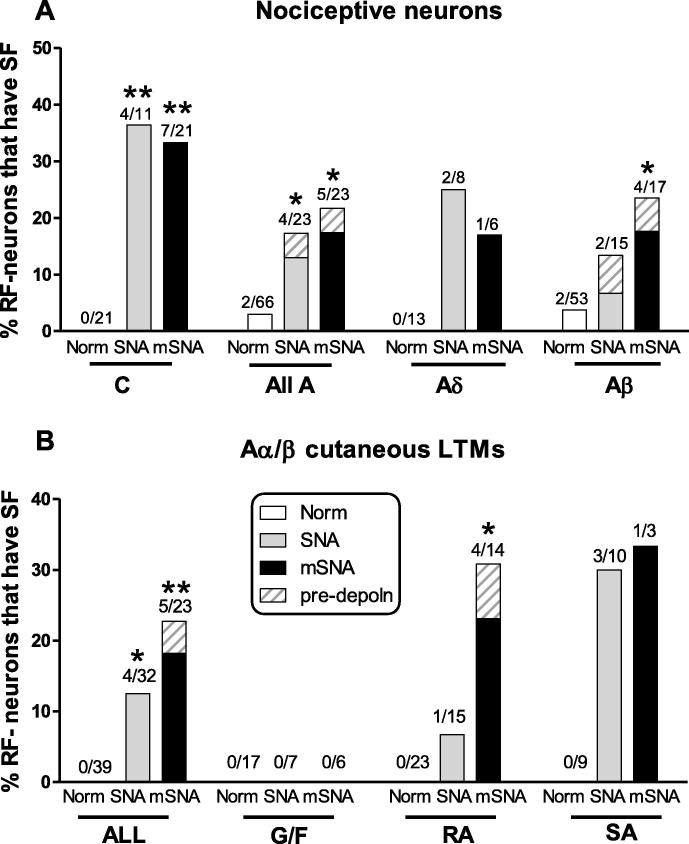
Spontaneous firing (SF): percentages of uninterrupted receptive field (RF)-neurons that showed SF (A) nociceptors and (B) Aα/β cutaneous low-threshold mechanoreceptors (LTMs). For each data set, the normal group was compared with 1) spinal nerve axotomy (SNA) and 2) modified SNA (mSNA) groups with 2 × 2 contingency tables with Fisher’s exact test. (A) Significant increases occurred in C- and A-fibre nociceptors in both models (includes identified nociceptors with receptive fields from [21] plus novel data, see text). (B) Percentages of all Aα/β cutaneous LTMs with SF increased significantly in both models. They increased in rapidly adapting (RA) units only in SNA, but no G hair/Field (G/F) units had SF in either model. The solid grey and black bars are units with SF action potentials arising from a flat baseline, suggesting a fibre origin for the SF; the cross-hatched areas indicate those with SF arising from a predepolarisation, suggesting a soma origin.

SF (ongoing stimulus-independent firing) was recorded for >1 minute prior to sensory testing to avoid any influence of natural search stimuli. Neurons with at least one spontaneous AP during this time, were classed as having SF. Short-lasting (a few seconds) injury discharge due to electrode impalement was excluded. This study refines and extends our previous study [21] in which C- and Aδ-nociceptive-type groups included both identified nociceptors, and units without identified RFs, that were classed as nociceptor-type neurons on the basis of their AP shapes that resembled those of nociceptors [30]. Percentages of neurons with SF presented here include only RF-neurons directly identified from their RFs as nociceptors or low-threshold mechanoreceptors (LTMs). In Fig. 3A, these include: 1) nociceptors that were included in the previous study (excluding the non-RF-neurons) [21]; these make up 60% of the C-fibre neurons (similar percentages for normal, SNA, and mSNA), and 70% of the A-fibre neurons; plus 2) subsequently recorded nociceptors with identified RFs. Only Fig. 3A contains any previously published data. All SF data for Fig. 3B (Aα/β cutaneous LTMs), plus *all* other data in the paper are previously unpublished. The advantages of presenting SF data only for RF-neurons are 1) exclusion of neurons that are axotomised, or damaged to the extent that they fail to conduct (for importance of this, see Section 2.6.3); and 2) identification and exclusion of cooling-sensitive neurons, since firing of unidentified cooling-sensitive terminals at room temperature could be mistaken for SF.

Spontaneous APs arising from predepolarisations (depolarisations leading into the spontaneous APs) suggest a soma origin for the firing; in contrast, spontaneous APs arising from a flat baseline suggest a fibre origin. Furthermore, low amplitude (∼1–3 mV) Em oscillations have previously been reported to give rise to APs or to be associated with AP generation in some neuronal somata in acutely excised DRG in vitro [5]. To determine the likely origin (soma or fibre) of spontaneous APs, we a) examined Ems at 1–2 mV resolutions before spontaneous APs; b) examined whether there were consistent predepolarisations; c) examined whether spontaneous APs arose from, or in phase with, any such oscillations; and d) averaged spontaneous APs by aligning their AP peaks, to determine whether they were time-locked to any oscillations.

### 2.6 Sensory receptive properties

Hand-held stimulators were applied to the left hind limb and flank to search for RFs. Nonnoxious stimuli including stroking the skin with a brush, light pressure, and tap with blunt objects were always applied first. Then, for neurons that failed to respond to these low-intensity stimuli, noxious mechanical and thermal stimuli were applied using fine- or coarse-toothed forceps, sharp objects (eg, needles), and heat (hot water at >50°C). The sensory receptive properties (sensory modalities) of DRG neurons in all groups of rats were classified as described previously for guinea pig [19,48] and rat [28,30]. Only neurons with identified RFs were included. A brief description of the different groups follows.

#### 2.6.1 Nociceptors

Nociceptors had C-, Aδ-, or Aα/β-fibres (abbreviated to C-, Aδ-, or Aα/β-nociceptors). They included: 1) high-threshold mechanoreceptive (HTM) units that responded only to noxious mechanical stimuli or had deep (subcutaneous) mechanical RFs. The latter were not tested with thermal stimuli, because the high thermal insulation of keratinised epidermis means that external thermal stimuli result in very steep thermal gradients, and thus, tissue and nerve fibre damage would result from externally applied thermal stimuli extreme enough to excite nociceptive fibres with deep RFs. 2) Mechano-heat units with superficial or dermal RFs that responded to noxious mechanical stimuli and also promptly to a single application of noxious heat; and 3) Aβ-nociceptors (see [24]); these included HTMs and moderate pressure (MP) nociceptors. MP-nociceptors responded weakly to moderate pressure (ie, pressure applied with a blunt object to the RF with an intensity greater than light pressure but not noxious or painful), and more strongly to noxious mechanical stimuli, encoding the stimulus intensity through the noxious range [14], as do other Aβ-nociceptors, see also [24] for review. MP-nociceptors were distinguishable from other Aβ-nociceptors, whether normally or in RF-neurons after nerve injury.

C- and A-fibre HTMs were classified as a) “superficial cutaneous” if they responded to needle pressure and pinching of superficial skin with fine number 5 forceps while lifting it away from the underlying tissue; these RFs were probably epidermal or immediately subepidermal (see [30]); b) dermal RFs required noxious stimulation (squeezing) of a fold of skin including the dermis; c) “subcutaneous or deep” HTMs were activated only by squeezing or strong pressure to muscles, joints, or deep fascia.

#### 2.6.2 Cutaneous Aα/β low threshold mechanoreceptors (LTMs)

Cutaneous Aα/β LTMs included: 1) Guard (G) hair follicle afferent units that responded to movement of one or more G hair (G), and Field (F) units that responded to skin contact or movement of a group of hairs [14]. These were grouped together as G/F units; 2) rapidly adapting (RA) units that responded to rapid movement of low-intensity mechanical stimuli across glabrous skin (thought to innervate Meissner corpuscles), to movement of claw, and units sensitive only to light tapping and/or 100–250 Hz vibration (possible Pacinian corpuscle units); and 3) slowly adapting (SA) units; these were discriminated from RAs by their sustained responses to sustained constant light pressure from a von Frey hair applied to the RFs. Because most muscle spindle afferents normally showed ongoing firing due to 1) stretch of the leg, and 2) presence of muscle relaxant [62], it was not possible to determine SF for this subpopulation of Aα/β-LTMs. They were therefore not included in this study.

Both MP-nociceptors and other Aβ-nociceptors fire more enthusiastically to pinch, prick, or squeeze noxious mechanical stimuli than to low-intensity stimuli. This is not the case for LTMs, including Aα/β LTMs (SA, RA, and G hair/Field units). These groups are clearly distinguishable from each other normally, and in SNA or mSNA.

#### 2.6.3 Reasons for including only neurons with identified receptive fields (RF-neurons)

Unidentified neurons may include axotomised neurons. It is important to include only RF-neurons because DRG neurons in vivo with uninterrupted conducting fibres exhibit changes that either do not occur in nonregenerating axotomised neurons (eg, increased SF in C-neurons [13,21,50,51,77]), or occur in the opposite direction (eg, decreased AP rise time) to nonregenerating axotomised, DRG neurons [53,69,80]. These differences probably result from axotomised/damaged neurons and uninterrupted neurons having, respectively, much decreased or greater access to target/peripherally derived trophic factors and inflammatory mediators [34]. Since 20%–40% of L4 DRG neurons were axotomised/damaged after L5 SNA [20], the chances of contamination of the L4 data set with damaged neurons is high unless only RF-neurons are included.

The above absence of C-fibre SF refers to damaged/axotomised L5 C fibre after L5 spinal nerve injury (SNL/SNA) in which regeneration is not possible; C-fibre SF has been reported in other models in which regeneration may occur (chronic constriction injury or crush) [33,40,78].

#### 2.6.4 Limitations of sensory testing

Although sensory receptive properties were fully characterized as above, mechanical and thermal thresholds were not routinely measured. In this in vivo preparation, all stimuli are applied to the exterior of the skin. Determining mechanical thresholds requires precise mechanical stimuli, for example, von Frey hair application at right angles to the sensitive part of the RF. The complex geometry of the foot, foot pads, toes, and leg, and inaccessibility of some RFs (beneath or medial to foot/leg) make it difficult for the thresholds to be accurately determined in most cases. In addition, mechanical stability is an issue. Precise determination of thresholds requires more stimuli to tissues; since these are in mechanical continuity with the DRG being recorded, this increases the risk of losing the recording, especially in C neurons.

### 2.7 Selection criteria and statistical tests

For behavioural tests, comparison of day 7 ipsilateral values with preoperative values, on the same rat, were made with paired tests. For normally distributed data (SFL), paired *t*-tests were used, and for any variables that were not normally distributed (mechanical-hypersensitivity/allodynia and heat-hypersensitivity/hyperalgesia), Wilcoxon matched-pairs signed-rank tests were used. Mann-Whitney tests were used to compare, for each behaviour, SNA with mSNA treatment groups and SNA with mSNA pretreatment groups.

All electrophysiological data were from neurons with Ems of −40 mV, or more negative, that had overshooting-evoked APs and, for A-fibre neurons only, afterhyperpolarisations. All groups included recordings with KCl-filled electrodes; for SF and Em only, additional data were included from recordings with electrodes filled with LiCl with Lucifer Yellow (see earlier).

Percentages of uninterrupted/conducting RF-neurons in SNA and mSNA rats with SF were compared with those of normal neurons using 2 × 2 contingency tables and Fisher’s exact test (Fig. 3). Nonparametric statistics were used for comparison of electrophysiological variables. For each CV group and each group defined by its sensory properties, medians of two groups were compared with Mann Whitney tests (Fig. 5), and between three groups were compared using Kruskal-Wallis tests, with Dunn’s post tests comparing 1) SNA with normal rats and 2) mSNA with normal rats (Figs. 6 and 7). Significance for all tests (Figs. 2, 3 and 5–7) is indicated as ^∗^*P* < 0.05, ^∗∗^*P* < 0.01, ^∗∗∗^*P* < 0.001, ^∗∗∗∗^*P* < 0.0001. All tests were made with GraphPad Prism 5 software (GraphPad Software Inc, La Jolla, CA, USA).

## 3 Results

### 3.1 Pain behaviour

Pain behaviour values 7 days after SNA/mSNA induction were compared with preoperated (Pre) values. Spontaneous foot lifting (SFL), absent normally, was increased highly significantly 7 days after mSNA, but showed only a very small (not significant) increase (<1 second in 10 minutes) in SNA (Fig. 2A and B), as shown previously [21]. The paw withdrawal threshold to von Frey hair stimulation decreased significantly in SNA, but decreased more in mSNA rats (very highly significant) (Fig. 2C and D). Paw withdrawal latency to heat (Hargreaves test) was significantly reduced in SNA rats, but reduced more, and with greater significance, in mSNA rats, showing greater heat hypersensitivity in mSNA. All 3 behaviours, spontaneous and evoked, both mechanical-hypersensitivity (allodynia) and heat-hypersensitivity (hyperalgesia), were significantly greater in mSNA than SNA (Fig. 2), but there were no pretreatment differences between SNA and mSNA groups for any of the behaviours.

To explore possible mechanisms underlying these behavioural changes, we examined the electrophysiological properties of L4/L5 DRG neurons in normal and L4 RF-neurons in SNA and mSNA rats.

### 3.2 Electrophysiology

Recordings were made from L4/L5 DRG neurons in normal (untreated) rats (n = 55) and from ipsilateral L4 RF-neurons in 9 SNA and 18 mSNA rats 7 days postoperatively. The numbers of neurons in each CV and sensory group after different treatments are indicated on the figures. The numbers of units differ for each variable recorded, because not all recordings provided all types of data seen earlier (minimum recording time needed for SF, AP overshoot needed for AP rise time, only some neurons tested for threshold, and neurons with LiCl-filled electrodes were included only for threshold and Em). In the following sections, changes in RF-neurons are described first for nociceptors and then for cutaneous Aα/β-LTMs. C-LTMs and Aδ-LTMs, which normally constitute a low percentage of normal neurons recorded [30], were not encountered in SNA and mSNA rats.

#### 3.2.1 Spontaneous firing (SF) in L4 RF-neurons

##### 3.2.1.1 Nociceptors

None of the L4 C-nociceptors (which excluded C-cooling and C-mechano-cold units) in normal rats showed SF. There was a significant increase in percentages of C-nociceptors with SF in SNA rats to 36.4% and in mSNA rats to 33.3% (Fig. 3A). Most C-nociceptors with SF were C HTM units with deep (subcutaneous) RFs (C HTM deep) whose response to thermal stimuli could not be tested due to RF depth. Types of C-nociceptors with SF were as follows: in SNA: 4 C HTM deep units; in mSNA: 4 C HTM deep units, 2 C mechano-heat units, and 1 dermal C HTM unit. This suggests that SF arises in C-nociceptors with RFs at different depths in the tissues, at least some of which are thermally sensitive.

The spontaneous APs in C-fibre neurons recorded at Ems of −40 mV or more hyperpolarised, all arose from a flat baseline. SF arising from a flat baseline, and/or not associated with soma membrane oscillations, is likely to originate outside the soma. Although the presence or absence of Em oscillations in most neurons was usually unequivocal, about half of C-fibre neurons showed some possible low amplitude (<1 mV) oscillations that were not associated with the spontaneous APs. These observations in conducting L4 C-nociceptors (RF-neurons) differ from those in acutely excised DRG in vitro [5], where injured C-type DRG neurons without Em oscillations did not fire spontaneously. However, their C neurons would have been axotomised/injured; in our models we see no SF in the axotomised C neurons (see Section 2.6.3).

The lack of SF in axotomised C afferents in L5 spinal nerve injury models (see Section 2.6.3) means that their inclusion would decrease the percentage of neurons with SF. The similar percentages of identified C-nociceptors with SF reported here to those in our previous study [21], which included unidentified nociceptor-type C neurons, suggests that most of those unidentified nociceptor-type C neurons were nociceptors or silent (very high threshold) nociceptors.

Unlike percentages of neurons that showed SF, the median firing rate of C-nociceptor-type neurons (nociceptive plus unidentified) was significantly greater in mSNA than SNA rats [21]. In that paper, we did not distinguish between C-fibre nociceptive neurons (with RFs), cooling sensitive neurons, and those without RFs. We therefore give medians of SF rates for only the L4 nociceptive RF-neurons here. In impulses/second, they were 1.8 (n = 6) for mSNA and were significantly lower for SNA 0.3 (n = 4, *P* < 0.05, Mann-Whitney).

**Fig. 4:**
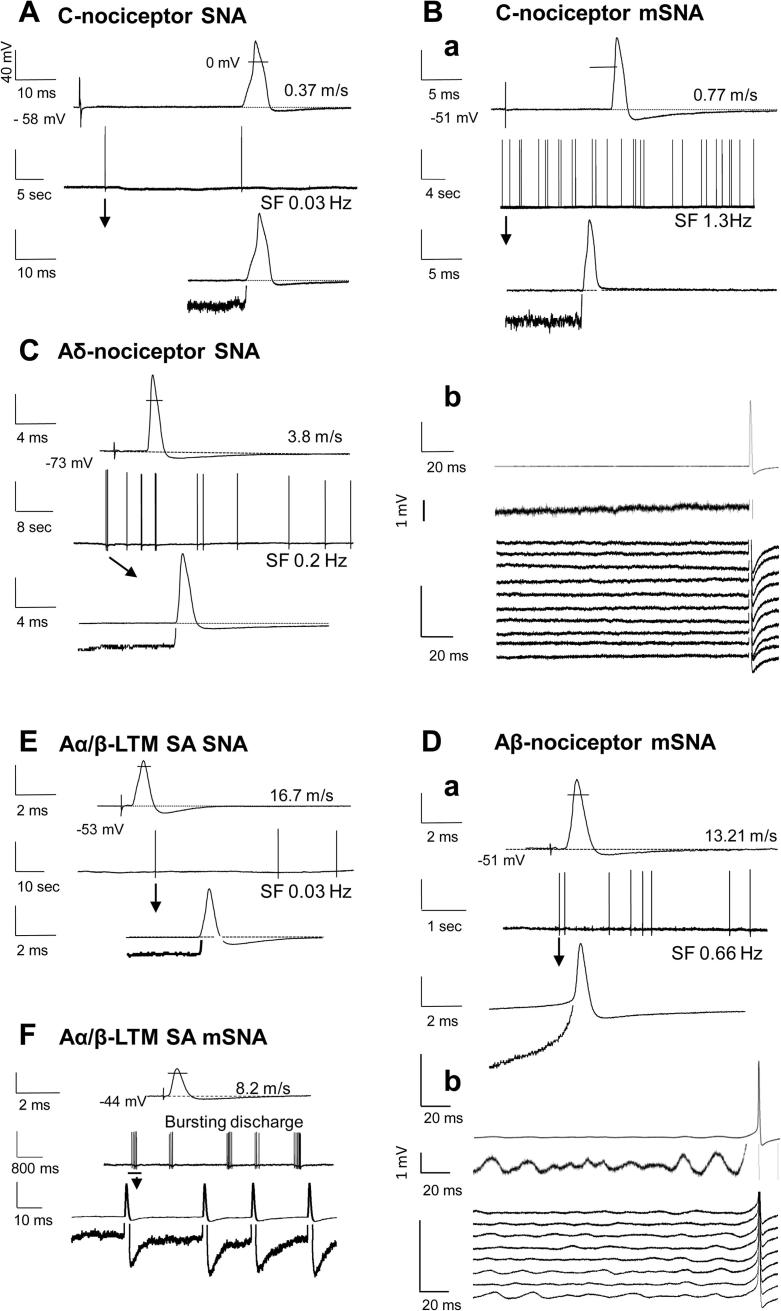
Representative examples of evoked and spontaneous action potentials (APs) from nociceptors and Aα/β-cutaneous low-threshold mechanoreceptors (LTMs). (A–F) show examples of intracellularly recorded APs. The 4 traces in A, Ba, C, Da, E and F were as follows. Line 1 (top): AP electrically evoked from the dorsal root. Line 2: Spontaneous firing (SF) over an extended time; SF firing rate shown below line for (A–E). Line 3: the first spontaneous AP shown in line 2. Line 4 (bottom): the same trace and same time scale as line 3 to show the leading edge of the AP at 8 times higher vertical resolution, truncating the AP. For all neurons (A–F), unless otherwise labelled, vertical scales are 40 mV for lines 1–3, and 5 mV for line 4. In (Bb) and (Db), the top 2 traces are for averaged SF APs (15 APs for Bb and 8 APs for Db). The time scales are shown to the left of the traces. Neurons were L4 receptive field-neurons in spinal nerve axotomy (SNA) (A, C, and E) and modified SNA (mSNA) (B, D, and F). They were C-nociceptors (A and B), an Aδ-nociceptor (C), an Aβ-nociceptor (D), and an Aα/β LTM slowly adapting (E, F). The conduction velocity (m/s) and membrane potential (Em; −mV) for each neuron are shown. APs were overshooting; the small lines cross the evoked APs (lines 1) at 0 mV. Note the faster SF rate in C-nociceptor in mSNA (B) than in SNA (A). (A–E) show the predominant irregular pattern of SF. (F) is the only neuron with short-bursting irregular discharges. Spontaneous APs arise from a flat baseline in (A–C) and (E), indicative of a fibre origin (see text). This is shown in (Bb), where 15 spontaneous APs are averaged shown at different vertical scales: 40 mV (line 1) and 1 mV (line 2). The bottom set of traces of (Bb) are a waterfall plot of the individual APs (1 line for each of the 15 spontaneous APs averaged above) showing no evidence of Em oscillations in relation to the APs. In the Aβ-nociceptor in (D), the SF APs have a predepolarisation, suggestive of a soma origin; the averaged traces for 8 SF APs in (Db) show baseline oscillations prior to SF APs; in the waterfall plot (bottom traces Db), these are clear for 6 of the 8 APs. In (F), the burst SF in the slowly adapting (SA) neuron has no predepolarisations; and is not associated with membrane oscillations.

Percentages of A-nociceptors (excluding mechano-cold units) that showed SF increased from ∼3% normally to 17% in SNA and 22% in mSNA rats, with similar increases in Aδ- and Aβ-nociceptors (Fig. 3A). Types of A-nociceptors with SF were as follows: in normal rats: 2 Aβ HTMs superficial; in SNA: 2 Aδ HTMs superficial, 2 Aβ HTMs deep; in mSNA: 1 Aδ HTM deep, 3 Aβ HTMs deep (one of which had predepolarisations, see Fig. 4D), and 1 Aβ HTM superficial. Thus, SF was not restricted to superficial or deep cutaneous RFs. Most spontaneous APs in A-fibre nociceptors in SNA and mSNA rats clearly arose from flat Em baselines (example in Fig. 4C), suggesting fibre origins for the SF. The one exception was an Aβ-nociceptor (see Fig. 4D) in which all spontaneous APs arose from a predepolarisation suggesting a soma origin. Averaging of spontaneous APs recorded at resting Em of −51 mV in this neuron showed a tendency for at least 2 oscillations to precede the APs, although the waterfall plot in Fig. 4Db shows this to be more obvious in 6 of the 8 spontaneous APs. Some oscillations with greater amplitude than these, however, were not associated with spontaneous APs. Thus, the oscillation amplitude in the soma may not have been the only contributory cause of spontaneous AP generation. Interestingly, when the Em became (spontaneously) more hyperpolarised to −60 mV, the oscillations were at lower amplitude as described previously [5], and no SF was recorded. This is consistent with a soma origin of the firing in this one A-fibre nociceptor, as rates of fibre-generated SF should be independent of soma Em.

Only one of the Aβ-nociceptors classed as MP-nociceptors (12 units in normal rats, 1 in SNA and 1 in mSNA), showed SF, this was the mSNA unit; it showed a fast SF rate relative to other L4 A-nociceptors of >5 Hz, and arose from a flat baseline.

##### 3.2.1.2 Aα/β-low threshold mechanoreceptors (LTMs)

Normally, none showed SF (Fig. 3B) increasing significantly in both SNA (to 12.5%) and mSNA rats (to 23%). No G hair/Field units showed SF after nerve injury, but increased percentages of RA and SA units showed SF (Fig. 3B). Most Aα/β-LTMs (both RA and SA) with SF showed spontaneous APs that arose from a flat baseline, suggesting a fibre origin of the APs (Fig. 4). One, a Pacinian RA unit with an Em of −55 mV, showed clear signs of a soma origin of the SF, with clear predepolarisations (not shown). Generally, the SF was irregular, but in 1 neuron, the only SA Aα/β-LTM in an mSNA rat (shown in Fig. 4F), the firing was bursting in nature, with bursts of SF, usually about 4–6 APs (Fig. 4F). This neuron showed regular Em oscillations (not shown), but the first AP of each burst was not preceded by a predepolarisation. Furthermore, averaging of the first spontaneous AP in each burst showed no relationship between the onset of the AP and the Em oscillations. These observations suggest that these spontaneous APs were of fibre origin.

**Fig. 5:**
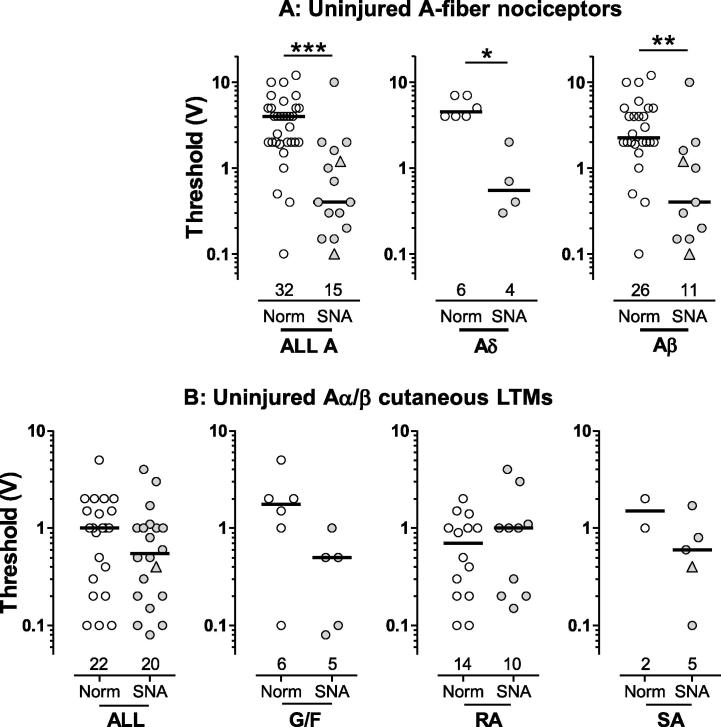
Dorsal root electrical thresholds: each symbol represents one neuron. All neurons had receptive fields (RFs). L4 and L5 DRG neurons in normal and L4 RF-neurons in spinal nerve axotomy (SNA) rats are plotted. Statistical tests were Mann-Whitney tests. The stimulus voltage (0.03 ms) at the dorsal root required to evoke a somatic AP is plotted as the threshold. Neurons with spontaneous firing are indicated as triangles. (A) In A-fibre nociceptors, thresholds were significantly lower than normal in SNA rats for all A-nociceptors, Aδ-nociceptors, and Aβ-nociceptors. (B) In Aα/β cutaneous low-threshold mechanoreceptors (LTMs), thresholds in SNA rats were slightly lower than normals (not significant) in all LTMs and in G hair/Field units. G/F, G hair/Field units; RA, rapidly adapting units; SA, slowly adapting units. Significance indicated as ^∗^*P* < 0.05, ^∗∗^*P* < 0.01, ^∗∗∗^*P* < 0.001.

**Fig. 6:**
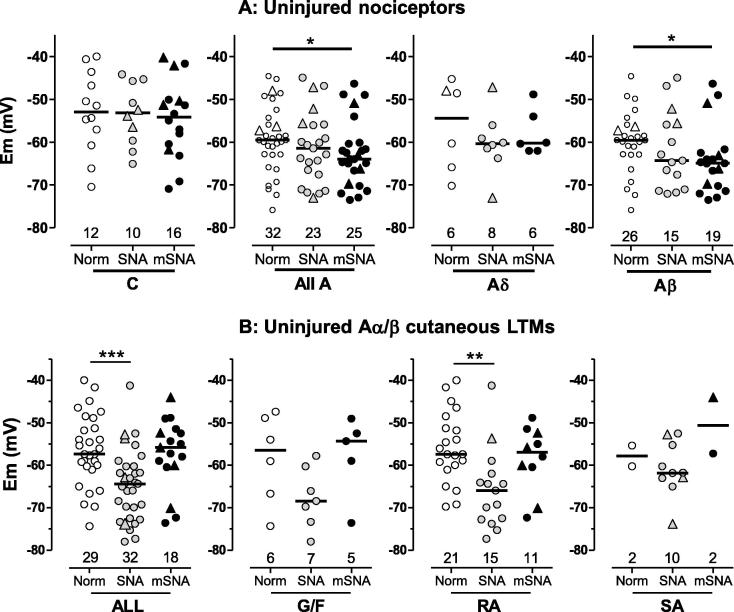
Membrane potential (Em). Scatterplots show distributions of Ems in L4 receptive field-neurons, in spinal nerve axotomy (SNA) and modified SNA (mSNA). (A) C- and A-nociceptors: Em was unchanged in C-nociceptors, but was hyperpolarised in A-nociceptors in SNA (not significant) and mSNA rats (significant). (B) Cutaneous Aα/β-low-threshold mechanoreceptors (LTMs): Em was significantly hyperpolarised in all cutaneous Aα/β LTMs together and in RA units in SNA rats, but no change from normal was seen in mSNA rats. For dorsal root ganglia recorded, statistics, and symbols, see Fig. 5 legend. G/F, G hair/Field units; RA, rapidly adapting units; SA, slowly adapting units.

**Fig. 7:**
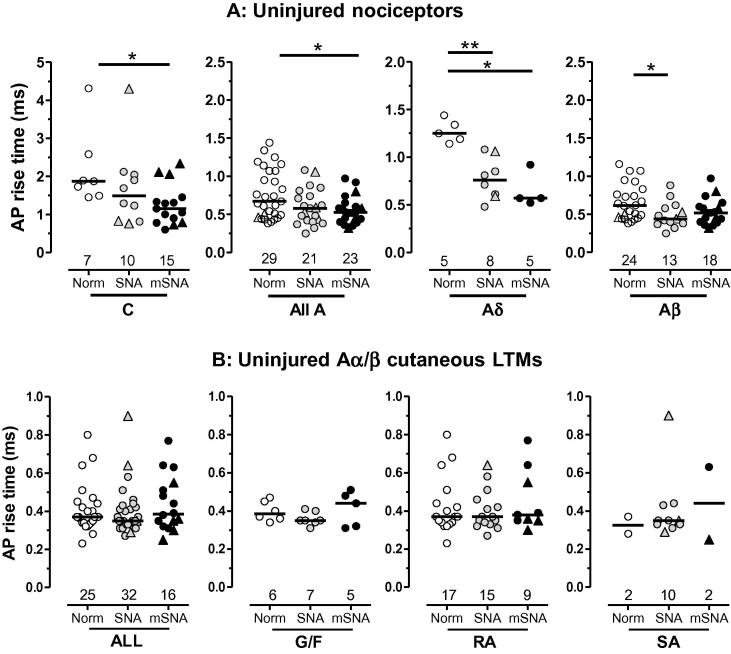
Action potential (AP) rise time. Scatterplots show distributions of AP rise time. (A) Nociceptors: compared with normal, AP rise time was significantly shorter in C-nociceptors in modified spinal nerve axotomy (mSNA), in all A-nociceptors (mSNA) and Aδ-nociceptors (SNA and mSNA) and in Aα/β-nociceptors (SNA). (B) Cutaneous Aα/β- low-threshold mechanoreceptors (LTMs): there was no significant change in AP rise time in the cutaneous Aα/β LTMs or their subtypes in SNA or mSNA models. For dorsal root ganglia recorded, statistics, and symbols, see Methods and Fig. 5 legend. G/F, G hair/Field units; RA, rapidly adapting units; SA, slowly adapting units.

RF-neurons with SF (triangular symbols in Figs. 5–7), whether nociceptors or LTMs, showed no obvious tendencies towards high or low values of thresholds, Em, or AP rise times in any group of neurons.

##### 3.2.1.3 SF incidence in L4 RF-neurons; comparison with published in vivo studies

Our findings of SF in L4 C- and A-fibre RF-neurons are consistent with previous in vivo studies with peripheral nerves in situ, which all also found significant percentages of L4 C-fibre neurons [21,77] and A-fibre neurons [13] with SF after L5 SNL or SNA. We excluded in vitro or ex vivo studies from the above comparison, because cutting nerve branches plus greater mechanical and/or stretch damage to fibres or to the fragile C-neuron T-junctions prior to recording would prevent conduction from the periphery to the soma during recording (eg, [53]). This would prevent 1) recording of SF arising in the periphery, 2) determination of RF types and thus, 3) recognition of RF-neurons from those with fibres damaged during L5 spinal nerve surgery. In some studies, loss of in vivo environment (eg, by superfusion) would remove or alter influences that may acutely trigger SF in vivo.

#### 3.2.2 Fibre or soma source of SF

With intracellular (soma) recordings, APs that originate in a fibre are likely to be recorded arising from a flat Em baseline, whereas those that arise within the soma (such as injury potentials on penetration, or due to depolarisation) have clear predepolarisations (see [4] and personal observations). We excluded all such injury firing/discharge that occurred immediately after penetration, and excluded that due to depolarisation by only classing firing as SF when the Em was equal to or more negative than −40 mV. After these exclusions, all C-nociceptor SF arose from a flat baseline (Fig. 3, see examples in Fig. 4), which suggests that SF in these neurons arose from the fibre not the soma.

In most Aα/β-neurons (both nociceptors and LTMs) with SF, the SF also arose from a flat baseline, suggesting a fibre origin. However, in a few, the SF had a predepolarisation (see Fig. 4D, for percentages see Fig. 3), suggesting a soma origin of SF in these few neurons.

#### 3.2.3 Dorsal root electrical threshold

Electrical threshold was examined because any decrease is likely to result in increased evoked activity in L4 RF-neurons, which would increase input to the CNS. Because too few thresholds were measured in mSNA neurons for useful comparison (2 Aδ- and 2 Aβ-nociceptors), only SNA threshold values are plotted in Fig. 5. Sensory thresholds were not measured (see Section 2.6.4).

##### 3.2.3.1 Nociceptors

Slowly conducting Aδ-nociceptors (<3 m/s) had much higher thresholds than Aδ-nociceptors with CVs ⩾3 m/s, but there were too few for statistical comparison. A-fibre nociceptors with CVs >3 m/s showed a profound highly significant decrease (by 90%, note the Y-axis log scale) in median threshold in SNA rats (Fig. 5A). In SNA rats, Aδ-nociceptors with CVs from 3–6.3 m/s, showed a threshold decrease by 88%, and Aβ-nociceptors showed a decrease by 82%. In normal rats, thresholds of Aα/β-LTMs were lower than those of Aβ-nociceptors (*P* < 0.001) and all A-nociceptors (*P* < 0.0001, Mann-Whitney tests). Interestingly, in SNA rats, the thresholds of A-nociceptors decreased to the extent that they were not significantly different from those of the cutaneous Aα/β-LTMs (Fig. 5A). Neurons with SF (triangles) did not have consistently lower or higher thresholds than those without.

##### 3.2.3.2 LTMs

In SNA compared with normal rats, the median electrical threshold for all cutaneous Aα/β-LTMs and for G hair/Field units appeared slightly lower (not significantly), with no change in RA units and not enough normal data for comparison of SA units (Fig. 5B).

#### 3.2.4 Membrane potential (Em)

Em can influence threshold and/or SF. We therefore explored somatic Em for the different neuronal subgroups of L4 RF-neurons.

##### 3.2.4.1 Nociceptors

Median Em was unchanged in C-fibre nociceptors in SNA and mSNA rats compared with normal rats (Fig. 6A). In contrast, in L4 A-fibre nociceptors, Em was similarly hyperpolarised in both SNA (*P* < 0.05) and mSNA rats (*P* < 0.001). Subdividing A-nociceptors into Aδ- and Aβ-nociceptors shows similar decreases for both groups in SNA and mSNA, although significant only for Aβ-nociceptors in mSNA rats (*P* < 0.001).

##### 3.2.4.2 LTMs

In SNA but not mSNA, cutaneous Aα/β-LTMs had a more hyperpolarised median Em (Fig. 6B). Similar changes within subtypes of these neurons were not significant in G hair/Field units, but highly significant in RA units.

Thus, median Em was hyperpolarised in A-fibre nociceptors and Aα/β-LTMs (in SNA rats), but not in C-nociceptors, suggesting different underlying mechanisms of Em control in C and A neurons.

#### 3.2.5 Action potential (AP) rise time

AP rise time was measured as described previously [25]. It is presented because decreased AP rise time is likely to indicate changes in Na^+^ inward current due to greater channel availability and/or activation, and thus could contribute to increased neuronal excitability.

##### 3.2.5.1 Nociceptors

AP rise time was decreased in L4 C-, Aδ-, and Aβ-nociceptors, but with slightly greater decreases in mSNA than SNA rats in C- and Aδ-nociceptors, but not Aβ-nociceptors (Fig. 7). These changes were significant in SNA for Aδ- and Aβ-nociceptors, and for mSNA in C- and Aδ-nociceptors and all A-nociceptors. Aβ-MP nociceptors were highly represented in the lowest 4 Aβ-nociceptor values in each column, 3/4, 2/4, and 2/4 in normal, SNA, and mSNA rats, respectively. This is not surprising because these MP-nociceptors have some of the fastest CVs [14,30], and because CV is related to somatic AP rise time, as well as to fibre diameter. In contrast to AP rise time, median AP fall time was not altered in any CV group of nociceptors (not shown).

##### 3.2.5.2 LTMs

AP rise time (Fig. 7B) and fall time (not shown) were unchanged in both SNA and mSNA rats in cutaneous Aα/β-LTMs.

#### 3.2.6 Conduction velocity (CV)

Median CVs did not differ for C- Aδ- or Aβ- or all A-nociceptors between normal and either SNA or mSNA rats. Furthermore, comparison of units with similar CVs between the different groups shows that in both C- and A-nociceptors, the AP durations are clearly shorter in the SNA/mSNA rats. Thus, the decrease in AP rise time was not due to selection of neurons with faster CVs in the SNA/mSNA rats.

## 4 Discussion

The altered electrophysiological properties in vivo of L4 RF-neurons after L5 SNA or mSNA would increase the afferent input to the CNS, and thus should contribute to NP. Changes, which include increased SF in C- and A-nociceptors and cutaneous Aα/β-LTMs, plus decreased electrical thresholds and hyperpolarised Em in A-nociceptors, may contribute to different aspects of NP.

### 4.1 The importance of in vivo receptive field (RF) identification

This is the first intracellular study in vivo that includes only L4 RF-neurons. This is important because nonregenerating axotomised L5 neurons differ markedly from L4 RF-neurons (see Section 2.6.3). Previous studies of L4 DRG neuronal somata after L5 SNL or SNA probably included axotomised neurons: they were in vitro (ie, with axotomy-like phenotypes [42]), acutely ex vivo and/or included unidentified neurons. L5 SNL/SNA surgery results in injury to the L4 spinal nerve [21,67]. This means that, since previous studies including ours [21] were not limited to neurons with RFs [53,63,80], they may well include axotomised neurons.

### 4.2 Spontaneous/ongoing firing (SF) in L4 RF-neurons

Our findings of 1) increased percentages of C-nociceptors with SF in SNA and mSNA rats and 2) greater/faster C-nociceptor SF rate in mSNA than SNA are consistent with our previous observations in C-nociceptor-type neurons in the same animal models of NP [21]. They further confirm the contrast between C-nociceptors with SF (∼35%) and axotomised C neurons with no SF (see [21] and Introduction), and between cutaneous Aα/β-LTMs with SF (∼20%) and axotomised cutaneous A-fibre LTMs with no SF [55].

#### 4.2.1 The site of origin of SF

A-fibre origin for SF in all C- and Aδ-nociceptors and in most Aα/β-neurons is suggested by SF APs arising from a flat baseline. Acute cut of the dorsal root precludes a CNS origin and did not cause SF in normal rats. The intact peripheral nerve is the more probable source because the sites of injury and inflammation (likely triggers of SF) are peripheral to the DRG. This interpretation is consistent with absence of SF in L4 C fibres following L5 SNL, after acute cut of the L4 spinal nerve [13]. However, the extent to which the SF originates in peripheral terminals or the fibre (perhaps at site/s of neuroinflammation) is, as yet, unclear, although both are consistent with the finding that lidocaine injection into RFs of 3 afferent C fibres after SNL blocked SF in 2 of them [77]. In contrast, a soma origin for SF in a few Aα/β-neurons is suggested by predepolarisations/Em oscillations (see Section 3). This may reflect greater membrane instability throughout those neurons, resulting in soma and/or fibre sites of SF origin.

#### 4.2.2 Possible causes of SF

Ongoing influences on uninterrupted afferent peripheral fibres in the SNA/mSNA models include target-derived trophic factors, plus neuroinflammation induced by degeneration of axotomised fibres (SNA/mSNA) and by the chromic-gut L4 ligation (mSNA only) [21]. The altered environment (eg, decreased pH, increased temperature) induced by neuroinflammation may increase SF likelihood through longer-term up- or downregulation of receptors/ion channels and acute activation/sensitization of receptors [44]. We give transient receptor potential V1 channel (TRPV1) as an example, because it is relatively well understood. Interestingly, TRPV1 in C fibres can be activated along the fibre, not just at terminals [37]. Transduction may thus occur at terminals or along uninterrupted C fibres, with increased likelihood of firing within region/s of neuroinflammation, due to threshold reduction and sensitization of thermal responses of TRPV1 by, for example, some inflammatory mediators and acid pH (see [70]). Thus, sensitization could result in ongoing firing in response to normal or slightly elevated temperatures; such ongoing firing would be characterized as SF. Other likely contributors include tumour necrosis factor-α, which causes SF [65], and other inflammatory mediators. Greater sensitivity to inflammatory mediators in small neurons [47] probably contributes to greater percentages with SF of C-nociceptors than A-neurons.

The finding that neurons with lowest thresholds or fastest AP rise times were not necessarily those with SF, may indicate that the ability to fire spontaneously differs between neurons with different chemical phenotypes (eg, expressing trkA or GFRα1/IB4-binding) and/or different ion channel complements (see [47]). The present relationships could therefore usefully be re-examined within such defined subgroups.

### 4.3 Membrane potential (Em)

Hyperpolarised Em in A-fibre neurons but not C-nociceptors suggests differing Em control mechanisms, which are not yet understood. Important ion channel candidates for Em control/influence include K^+^ leak 2-pore-domain (K2P) channels [27,41] and hyperpolarisation-activated (*I*_h_) channels [10,61]. Expression of some of these differs between neuronal subgroups, for example, certain K2P channels are differentially expressed in different-sized DRG neurons with high TWIK1 in large neurons and high TREK1 and TREK2 in small neurons [2,71], although their contribution to NP is not understood. *I*_h_ is most prominent in Aα/β-DRG neurons [38], and our studies (paper submitted).

### 4.4 AP rise time

In C- and Aδ-nociceptors, the shorter AP rise time in mSNA than SNA rats may result from cumulative neuroinflammatory influences from degeneration of axotomised fibres plus chromic-gut loose ligation of the L4 spinal nerve [21]. Shorter AP rise time in nociceptors suggests greater voltage-gated Na^+^ current density. This is probably related to the known inflammation-induced nerve growth factor (NGF)-dependent upregulation of Nav1.7 and Nav1.8 in nociceptors, and to their upregulation and increased Na^+^ current density in L4 DRG neurons after L5 SNL/SNA [13,32,59,80]. The normally lower expression of Nav1.7 and Nav1.8 in Aα/β-LTMs [20,26,29,76] and the lower/absent trkA expression and NGF influence on these neurons [29] may account for their lack of change in AP rise time.

### 4.5 Electrical threshold

In A-nociceptors, lowered electrical thresholds in SNA were accompanied by generally shorter AP rise time and hyperpolarised Ems. A combination of 1) increased Nav1.8 and Nav1.7 expression (see earlier) and 2) hyperpolarisation-induced increased Nav1.7 availability (which is normally only ∼10% but increases with hyperpolarisation [36]), probably contribute to the decreased thresholds. Reduction of Nav1.8 activation threshold by inflammatory mediators could also contribute (see [3]).

In Aα/β cutaneous LTMs, smaller or absent threshold change may result from their normally greater complement of low-threshold TTX-sensitive Nav channels and thus, lower electrical thresholds.

### 4.6 Possible contributions of electrophysiological changes in L4 RF-neurons to NP

#### 4.6.1 Spontaneous pain

Because C-nociceptor firing can cause slow burning pain, while A-nociceptor firing causes fast, sharp, pricking pain (eg, [35,57,72,73]), SF in C- and A-nociceptors probably result, respectively, in the ongoing/spontaneous unpleasant burning and sharp/shooting aspects of NP (see [35]). Greater SFL in mSNA than SNA is correlated with greater SF *rate* in C neurons in mSNA, not with the percentage of C neurons showing SF [21].

#### 4.6.2 Evoked pain

Mechanical thresholds of L4 A-nociceptors after L5 SNL reduce enough to contribute to allodynia as well as hyperalgesia [66]. The decreased electrical thresholds in A-nociceptors reported here could contribute to this reduced mechanical threshold, as follows. Because somatic Em and electrical threshold were measured some distance from the peripheral nerve injury/inflammation sites, they probably reflect changes in ion channel expression/activity throughout the neuron, including peripheral fibres. Lowered electrical thresholds in the periphery would enable APs to be triggered by smaller receptor potentials, thus decreasing sensory thresholds. The decreased electrical thresholds in A-nociceptors in SNA reported here were as low as those of normal cutaneous Aα/β-LTMs, and may therefore contribute to the previously reported nociceptor mechanical-hypersensitivity after L5 SNL [66] and to the mechanical allodynia reported here.

The heat-hypersensitivity we report may result from greater peripheral excitability in L4 neurons after L5 SNA/SNL due to increased Na^+^ channel activity (see earlier), and from inflammation-induced upregulation and/or sensitization of heat receptor molecules, such as TRPV1 (eg, [11,39,45]) (see Section 4.2.2).

As well as direct changes in nociceptors, sensitization of CNS neurons may contribute to the observed increased behavioural hypersensitivity. Because C-nociceptor activity is essential for triggering and maintaining central sensitization (see [16,46], the SF in C-nociceptors may be an important driver. Thus, the greater evoked behaviours in mSNA than SNA may relate to greater C-fibre SF rates in mSNA (this paper and [21]). It is unknown whether SF in L4 A-fibre nociceptors or Aα/β-LTMs also contribute to central sensitization.

#### 4.6.3 Paresthesias

SF in A-fibre LTMs is thought to contribute to paresthesias/dysesthesias (see [8]), but the neuronal subtypes showing SF were unknown. We show, for the first time, which cutaneous Aα/β-LTMs with uninterrupted fibres exhibit SF in models of NP: that is, RA and SA but not G/F LTMs. In patients with paresthesias, ectopic impulses in large myelinated fibres were suggested to result from lowered thresholds [58]. Whether the present SF is due to lowered thresholds and/or membrane instability is not clear from our data. However, the soma SF (indicated by predepolarisation) in a few Aα/β-LTMs may suggest Em instability. The SF in RAs could be related to the novel static firing reported in RA LTMs after nerve injury in rats [56].

In summary, we report changes in C- and A-nociceptors, and Aα/β-cutaneous LTMs that are consistent with the uninjured neuron hypothesis (see [15]), and that could contribute to different aspects of peripheral NP as follows: SF in C- and A-nociceptors to spontaneous burning and sharp-shooting pain, respectively; SF in Aα/β-cutaneous LTMs to paresthesias. Finally, if decreased A-nociceptor electrical thresholds contribute to sensory hypersensitivity, they would result in greater evoked pain (hyperalgesia and/or allodynia).

## Conflict of interest statement

Xin Fang is employed by Neuro Solutions Ltd. There are no other conflicts of interest.
