# Erratum to “Partial nerve injury induces electrophysiological changes in conducting (uninjured) nociceptive and non-nociceptive DRG neurons: Possible relationships to aspects of peripheral neuropathic pain and paresthesias” [Pain 153 (9) (2012) 1824–1836]

**DOI:** 10.1016/j.pain.2012.08.010

**Published:** 2012-11

**Authors:** Laiche Djouhri, Xin Fang, Stella Koutsikou, Sally N. Lawson

**Affiliations:** School of Physiology and Pharmacology, University of Bristol, Bristol BS8 1TD, UK

Due to an error during proofing some Greek symbols were omitted from the Abstract of this paper. The complete Abstract is provided below.

The Publisher apologises for this mistake.

## Abstract

Partial nerve injury leads to peripheral neuropathic pain. This injury results in conducting/uninterrupted (also called uninjured) sensory fibres, conducting through the damaged nerve alongside axotomised/degenerating fibres. In rats seven days after L5 spinal nerve axotomy (SNA) or modified-SNA (added loose-ligation of L4 spinal nerve with neuroinflammation-inducing chromic-gut), we investigated (a) neuropathic pain behaviours and (b) electrophysiological changes in conducting/uninterrupted L4 dorsal root ganglion (DRG) neurons with receptive fields (called: L4-receptive-field-neurons). Compared to pretreatment, modified-SNA rats showed highly significant increases in spontaneous-foot-lifting duration, mechanical-hypersensitivity/allodynia, and heat-hypersensitivity/hyperalgesia, that were significantly greater than after SNA, especially spontaneous-foot-lifting. We recorded intracellularly *in vivo* from normal L4/L5 DRG neurons and ipsilateral L4-receptive-field-neurons. After SNA or modified-SNA, L4-receptive-field-neurons showed the following: (a) increased percentages of C-, Aδ-, and Aβ-nociceptors and cutaneous Aα/β-low-threshold mechanoreceptors with ongoing/spontaneous firing; (b) spontaneous firing in C-nociceptors that originated peripherally; this was at a faster rate in modified-SNA than SNA; (c) decreased electrical thresholds in A-nociceptors after SNA; (d) hyperpolarised membrane potentials in A-nociceptors and Aα/β-low-threshold-mechanoreceptors after SNA, but not C-nociceptors; (e) decreased somatic action potential rise times in C- and A-nociceptors, not Aα/β-low-threshold-mechanoreceptors. We suggest that these changes in subtypes of conducting/uninterrupted neurons after partial nerve injury contribute to the different aspects of neuropathic pain as follows: spontaneous firing in nociceptors to ongoing/spontaneous pain; spontaneous firing in Aα/β-low-threshold-mechanoreceptors to dysesthesias/paresthesias; and lowered A-nociceptor electrical thresholds to A-nociceptor sensitization, and greater evoked pain.

